# Form-Finding Model Shows How Cytoskeleton Network Stiffness Is Realized

**DOI:** 10.1371/journal.pone.0077417

**Published:** 2013-10-17

**Authors:** Jinghai Gong, Daxu Zhang, Yiider Tseng, Baolong Li, Denis Wirtz, Benjamin William Schafer

**Affiliations:** 1 Department of Civil Engineering, Johns Hopkins University, Baltimore, Maryland, United States of America; 2 Department of Civil Engineering, Shanghai Jiao Tong University, Shanghai, China; 3 Department of Chemical Engineering, University of Florida, Gainesville, Florida, United States of America; 4 Department of Chemical and Biomolecular Engineering, Department of Oncology, Johns Hopkins University, Baltimore, Maryland, United States of America; Dalhousie University, Canada

## Abstract

In eukaryotic cells the actin-cytoskeletal network provides stiffness and the driving force that contributes to changes in cell shape and cell motility, but the elastic behavior of this network is not well understood. In this paper a two dimensional form-finding model is proposed to investigate the elasticity of the actin filament network. Utilizing an initially random array of actin filaments and actin-cross-linking proteins the form-finding model iterates until the random array is brought into a stable equilibrium configuration. With some care given to actin filament density and length, distance between host sites for cross-linkers, and overall domain size the resulting configurations from the form-finding model are found to be topologically similar to cytoskeletal networks in real cells. The resulting network may then be mechanically exercised to explore how the actin filaments deform and align under load and the sensitivity of the network’s stiffness to actin filament density, length, etc. Results of the model are consistent with the experimental literature, e.g. actin filaments tend to re-orient in the direction of stretching; and the filament relative density, filament length, and actin-cross-linking protein’s relative density, control the actin-network stiffness. The model provides a ready means of extension to more complicated domains and a three-dimensional form-finding model is under development as well as models studying the formation of actin bundles.

## Introduction

Eukaryotic cells are the building blocks of higher organisms. The cytoskeleton forms the internal framework of eukaryotic cells and is responsible for a cell’s elasticity and thus plays an important role in cell shape and motility. The mechanical behavior of the cytoskeleton is determined by the interactions of three types of filaments: actin filaments, intermediate filaments, and microtubules [1]. Actin filaments provide cells with primary mechanical support and are engaged as a driving force in cell motility [2].

The stiffness of the cytoskeleton network governs passive and active mechanical performance of cells. Many biological functions are intimately associated to the mechanical response, and therefore the cell’s stiffness may also serve as a sensitive indicator for the development or health state of a cell [2]. In recent years, numerous efforts have been made to predict the elasticity of the cytoskeleton network by using computational models; including open-cell foam models [3], tensegrity models [4-7], cable network models [8], lattice-based models [9], coarse-grained models [10,11] and Brownian dynamics models [12,13].

The pioneering work of Satcher and Dewey [3] employed a unit cell model of the cytoskeleton, in which the cross-linked actin filament network is treated as a solid matrix with random open pores (an open-celled foam) [2]. The model provides a simplified representation of the complex topology of the cytoskeleton in an average sense, and depends primarily on the bending stiffness of the actin filaments, and is independent of pre-stress forces in the network. Calibration to experimental data is required. The model represents a fine starting place for analytical exploration, but is limited in its ability to capture details of actual cytoskeletal networks.

Many researchers have employed tensegrity structures to describe the mechanical properties of a cell. Tensegrity is a structural principle based on the use of isolated compression elements inside a net of continuous tension elements with the aim of achieving a stable form in space [4]. Ingber and co-workers [14] first introduced the tensegrity concept to explain how cells and tissues are constructed and Stamenovic and Coughlin [8,15] have used a variety of tensegrity models from simple to complex to explore cytoskeletal networks. Stiffness of tensegrity models are dependent on the level of internal pre-stress, and opposite to open cell foam models, engage the axial stiffness of actin filaments and are independent of the bending stiffness of the actin filaments. Inclusion of pre-stress is important as experiments have established that cell stiffness is correlated with the level of pre-stress in both living [16-18] and reconstituted [19,20] actin networks. However, internal pre-stress in the network is a difficult quantity to measure, varies greatly across experiments [1] and results in significant uncertainty in the application of the model. In addition, while tensegrity does provide a stable three-dimensional cable configuration it is a very specific class of a much broader set of three-dimensional structures and little topologic evidence exists for its exact use in the cytoskeleton. Instead, much like the open-cell foam models it must be recognized as an intriguing starting point, particularly for analytical exploration, but is limited in its ability to capture many details of actual cytoskeletal networks.

A feature of all of the aforementioned models is that they have regular geometries and topologies. However, the topology of real cytoskeleton networks is highly diverse and complex. Randomness is a fundamental nature of cytoskeleton networks and should not be ignored. Attempts to introduce this randomness into cytoskeletal network models have been completed in tensegrity models [6] and through Monte Carlo simulation [11]. Another feature of developed computational models is that they are coarse-grained; that is, the true cytoskeleton system has far more filaments than the structural components in these models. Bausch and Kroy [21] proposed a multi-scale modeling approach to infer mechanical properties of filaments at the atomic scale and translate these into coarse-grained models of large filaments or filament networks [11]. Brownian dynamics models developed by Kim et al. captures broad features of the cytoskeleton network and are well-suited to describe the mechanical response in vitro; however the shear modulus (G') calculated by such models is small and efforts are still needed for accurate in vivo simulations.

In this work, our objective is to demonstrate the possibility of designing more complex and topologically relevant actin-network models using an equilibrium form-finding method. The paper addresses the development of the form-finding model, the components of which are generated by using a fully stochastic approach. The model is used to predict experimental observations of filament re-orientation, and study effects of the filament relative density, the filament length, and the relative density of filament cross-linkers on elastic modulus of the filament network.

## Methods and Models

Existing computational models for actin-networks are typically coarse-grained, drastically simplify and regularize the actual network topology, and require artificial external calibration. The objective of the form-finding model developed herein is to provide a fine scale model that is topologically consistent with actual actin-networks, does not require external calibration, and provides a means to directly explore the role of actin-network components, e.g. the influence of the average length of actin filaments on the elastic modulus of the network.

**Figure 1:**
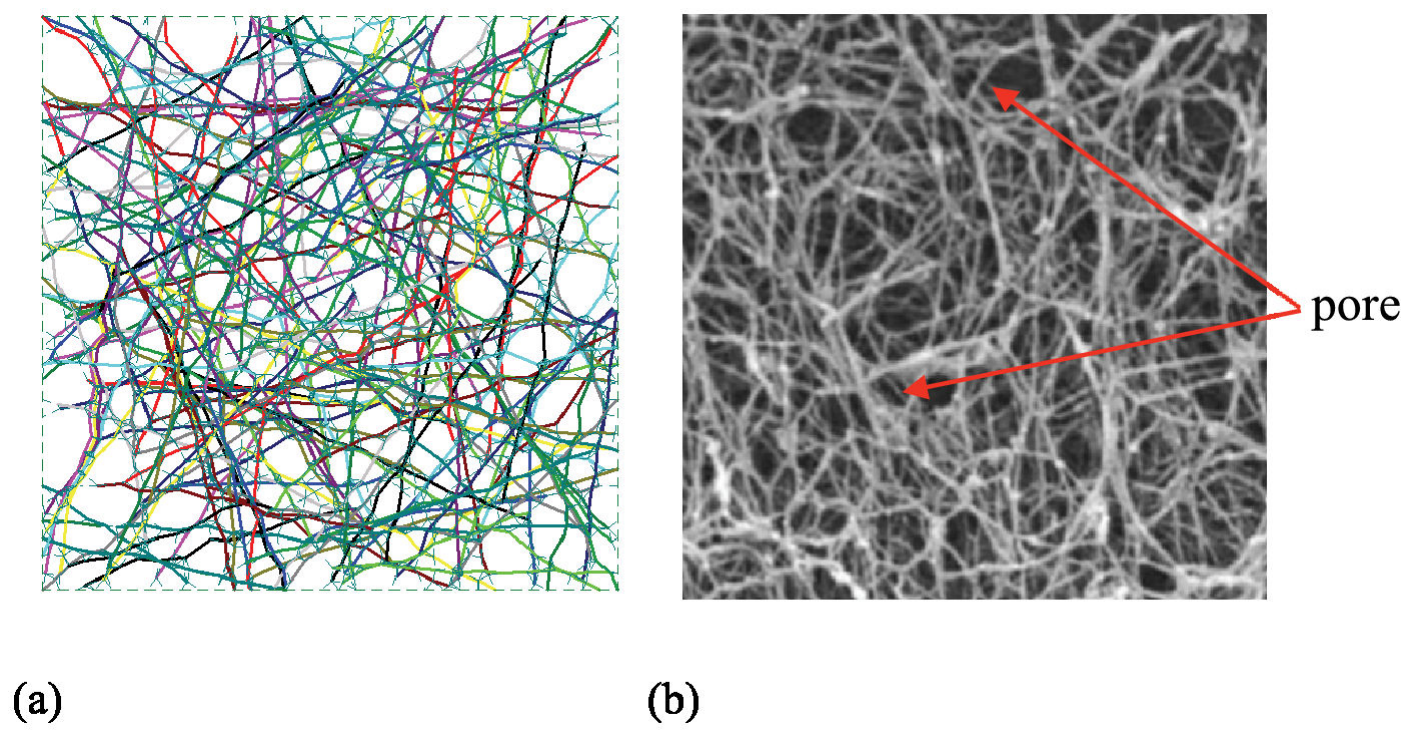
Typical actin-network (a) form-finding model (b) scanning electron micrograph.

To achieve this modeling objective, the equilibrium form-finding concept of structural/architectural engineering has been employed to find the natural form of cytoskeleton networks. Form-finding investigates how inter-connected networks deform into stable flexible structures. In structural/architectural engineering, form-finding may be used to find stable configurations of members for complex roofs and building structures. In nature, form-finding may be employed to discover how seemingly dis-organized structures are organized under mechanical stimulus. Form-finding analysis can be used to disclose the organizational principles of patterning in nature. In the mechanical sense, form-finding is the process of finding a stable equilibrium configuration for an arbitrarily defined initial system within a set of boundary conditions for a particular material or inter-connected components. Based on form-finding principles, models of actin-networks have been developed in this paper. Figure 1 shows a comparison between the current two-dimensional form-finding model and a scanning electron micrograph (SEM) of a typical actin-network. It can be seen that the form-finding model has the ability to provide a topologically realistic representation of the geometry of a real cytoskeleton.

### Model construction

#### Initial conditions

**Figure 2:**
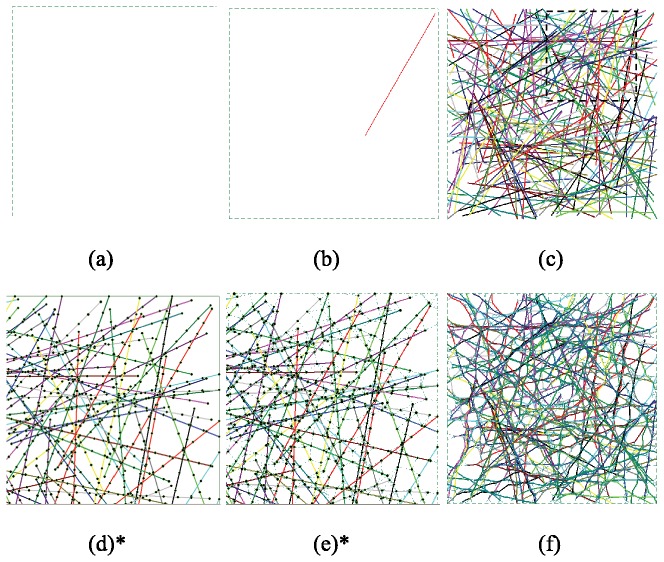
Generation of a filament and cross-linked network (a) a prescribed square domain (b) the first actin filament placed (c) actin filaments reaching the specified relative density (d) actin filaments are divided into segments by nodes (e) the network of actin filaments connected by cross-linkers (i.e., actin-cross-linking proteins) before form-finding analysis (f) the network of actin filaments connected by cross-linkers after form-finding analysis. *d and e show magnifications of the square region marked by the black dashed line in c.

Before creating a form-finding model, it is essential to determine a domain. In this case the domain defines the extent of the initial cytoskeleton network. As shown in Figure 2(a), a square domain has been chosen as the periodic representative element. Key length scales in the domain include the actin filament length and the domain side length. Obviously, the domain dimension has to be greater than the maximum actin filament length, but it is desirable to limit domain size in order to minimize the computational cost since a large number of stochastic simulations are necessary to study the relation between the network’s stiffness and the properties of its constituents. Based on these considerations and typical filament length’s reported in the literature (e.g., [22]) an average actin filament length of 5 μm is selected along with a square domain of 10 μm × 10 μm. 

#### Model parameters

The form-finding and actin-network stretching analyses are carried out with large deformation nonlinear finite element analysis. Beam and cable elements are used to model the actin filaments and their cross-linkers, respectively. Geometric and material properties for the network are based on available experimental measurements and are listed in Table 1. 

**Table 1:** Dimensions and material properties of actin filaments and cross-linkers.

Parameters	Data
Elastic modulus of actin filaments	1.4 GPa [30]
Diameter of actin filaments	7 nm [30]
Length of actin filaments	5 ± 2 μm [17]
Length of actin filament segments ^1^	0.3 ± 0.06 μm
Relative density of actin filaments ^2^	0.15% ~ 0.3% [1]
Yield tensile force of actin filaments	0.25 nN [31]
Maximum length of cross-linkers	0.3 μm [19]
Yield tensile force of cross-linkers	60 pN [18]

^1^ The maximum length of cross-linkers is used to achieve an accurate prediction.

^2^ Relative density is the amount of actin filaments per unit volume of filament network.

#### Actin filament generation

To generate the geometrical model, actin filaments are placed sequentially within a prescribed two-dimensional space, as shown in Figure 2(a). For each actin filament, length is sampled from a truncated (negative values are thrown out) Gaussian distribution with mean and standard deviation as reported in Table 1.Centroidal coordinates and angle of orientation of the filament are determined from uniform random distributions that cover the domain (X ~ 0-10 μm, Y ~ 0-10 μm, θ ~ 0-2π). If the actin filament falls out of the prescribed domain it is translated into the domain. An example of the first actin filament generated is provided in Figure 2(b). Additional randomly generated actin filaments are placed into the domain until relative density in the domain reaches a specified value. Figure 2(c) illustrates the layout of saturated actin filaments in a square domain, where actin filaments are represented by different colors.

#### Cross-linker generation

The modeled cytoskeletal network consists of an actin-network (of beams) cross-linked by proteins modeled as cables. Either α-actinin or filamin cross-linkers may be appropriate [23-26] here the model parameters (Table 1) are essentially aligned with filamin properties, but the stiffness of α-actinin may in the long term make it a more appropriate choice. At this stage, the goal is to demonstrate the model potential, but further detailed work on the cross-inker parameters is needed. Cable elements are used as cross-linkers to connect the actin filaments. The cross-linkers are generated in two steps. First, actin filaments are divided into segments to present their periodic binding sites to the cross-linkers as shown in Figure 2(d). Hence, cross-linkers may only attach at the binding sites (segment ends). The binding sites are selected such that they match expected pore size (see Figure 1(b)). Second, cross-linkers are created by connecting any two binding sites where the distance apart is less than the maximum cross-linker length. The resulting two-dimensional network model is provided in Figure 2(e) and is thus prepared for the form-finding step.

#### Form-finding

After a model such as that shown in Figure 2(e) is generated, a form-finding analysis is carried out to compute the final equilibrium shape of the actin-network. A small tensile pre-stress force (~3 pN or 5% of the tensile yield strength of the cross-linkers, Table 1) is applied to the cross-linkers to mimic the force state of an actin-cross-linking protein after it establishes a link between two actin filaments; and then a nonlinear finite element form-finding analysis is performed to compute the self-equilibrium configuration. During the form-finding analysis, the pre-stress force in the cross-linkers is kept constant. The small constant pre-stress force in the cross-linkers is only to facilitate the form-finding analysis. The pre-stress magnitude can be calibrated by comparing the predicted filament fluctuations with experimental observations. Preliminary studies of the cross-linker pre-stress magnitude indicate the expected stiffness varies by about 5% between minimal pre-stress and a pre-stress equal to the tensile yield strength of the cross-linkers. Figure 2(f) provides typical form-finding results for a cytoskeleton network, including actin filaments connected by actin-cross-linking proteins. The resulting networks share a strong resemblance with the known topology of actin filament networks such as shown in Figure 1(b) or the EM images of [27].

#### Effective modulus determination

**Figure 3:**
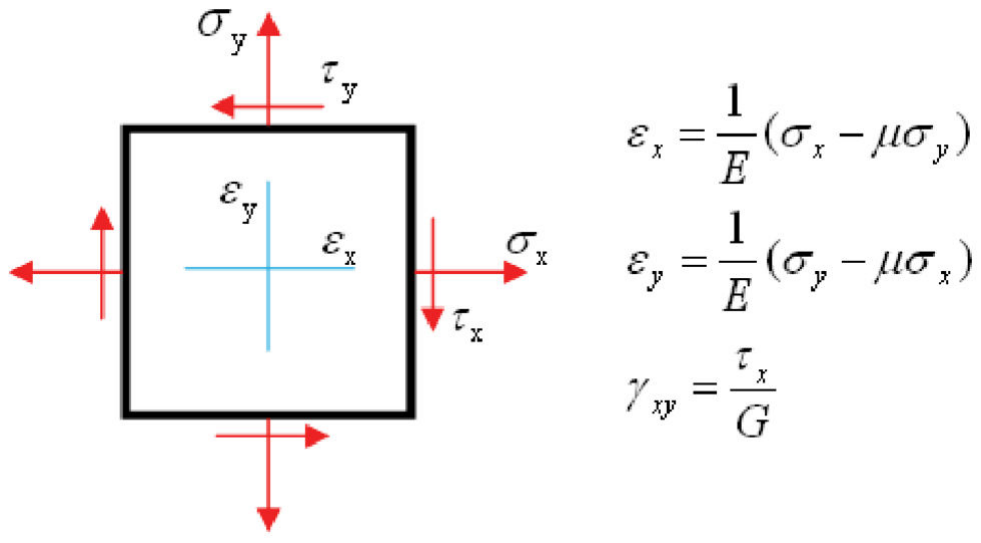
Schematic diagram of Hooke’s law for plane stress state.

After form-finding the developed representative volume element (e.g. Figure 2(f)) can be exercised to determine effective properties of the cytoskeleton under mechanical stimuli. The simplest of which is the effective elastic (Young’s) modulus. Elastic modulus is determined by the results from a simple extension of the model. One side of the domain is fixed in longitudinal translation while the far side is displaced longitudinally (*x* direction) a finite amount. Assuming plane stress conditions (Figure 3) for any subdomain in the model the effective elastic (Young’s) modulus is calculated by Hooke's law as$E=\left(\sigma_{x}^{2}-\sigma_{y}^{2}\right)/\left(\sigma_{x}^{}\varepsilon_{x}-\sigma_{y}^{}\varepsilon_{y}\right)$. For the simplest case where the subdomain studied is the entire model the effective engineering strain (ε_x_) is the finite stretch divided by the domain length (10 μm). The effective engineering stress (σ_x_) is the force required to create the finite stretch divided by the original cross-sectional area of the domain (10 μm x 1 μm) and σ_y_=0.

### Modeling sensitivity to sample and domain size

To obtain an optimum balance between computational cost and accuracy, modeling sensitivity to sample and domain size is assessed before completing further analysis. 

#### Modeling sensitivity to sample size

**Figure 4:**
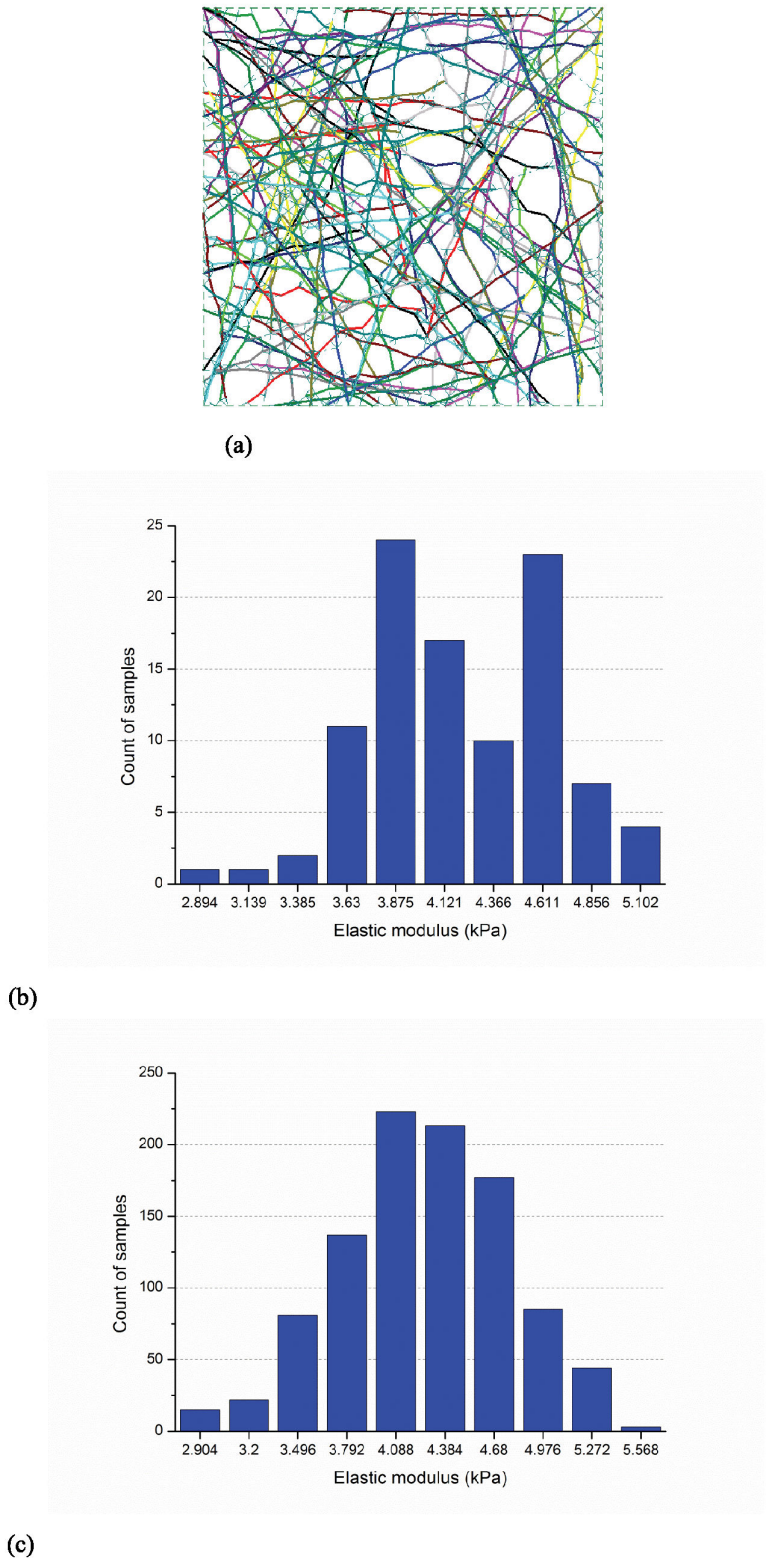
Typical model and impact of number of simulations (actin filament relative density: 0.2%, size: 10μm×10μm, Actin filament length: 5μm±2μm, segment length 0.3μm±0.06μm, and maximum cross-linker length: 0.3μm) (a) layout of a selected sample (b) histogram of elastic moduli for 100 samples: $\bar{E}$=4.38kPa, std(E)=0.47kPa (c) histogram of elastic moduli for 1000 samples: $\bar{E}$=4.41kPa, std(E)=0.52kPa.

Elastic modulus of networks with an actin filament relative density of 0.2% was determined from sample sizes of 100 and 1000 realizations of the 10 μm × 10 μm domain. A typical sample is provided in Figure 4(a) and histograms of the developed elastic moduli are provided for 100 samples in Figure 4(b) and 1000 samples in Figure 4(c). The larger number of samples provides a smoother approximation of the resulting probability distribution function (PDF) and better explores the extremes of the distribution, but the means, 4.38 kPa and 4.41 kPa, are very close. Since for the studies here mean elastic moduli is the main concern, the smaller sample size is deemed adequate.

#### Modeling sensitivity to domain size

**Figure 5:**
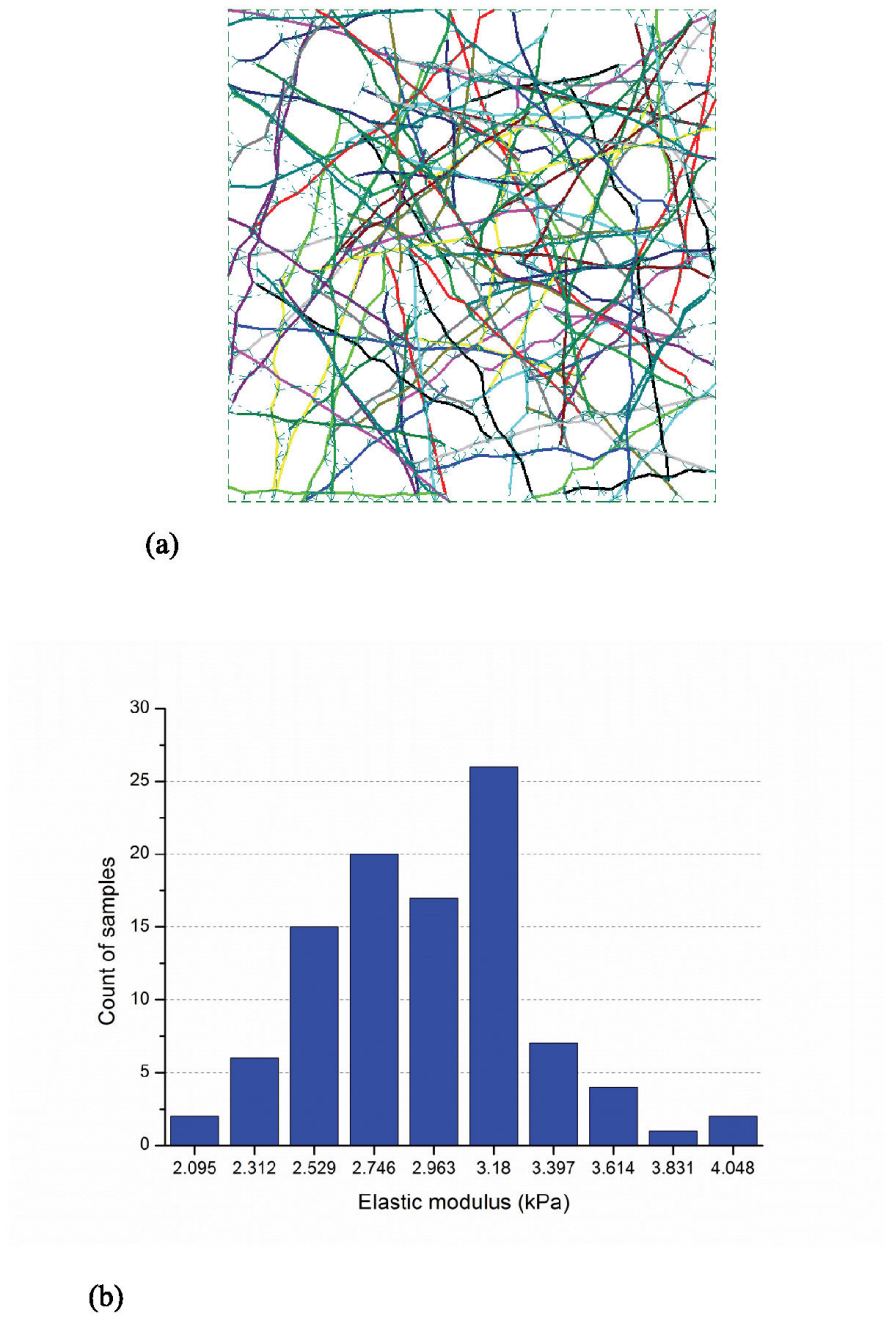
Typical model and response on 10μm×10μm domain (100 samples, actin filament relative density: 0.15%, actin filament length: 5μm±2μm, segment length 0.3μm±0.06μm, and maximum cross-linker length: 0.3 μm) (a) layout of a selected sample (10μm×10μm) (b) histogram of elastic moduli, $\bar{E}$=3.11kPa, std(E)=0.40kPa.

**Figure 6:**
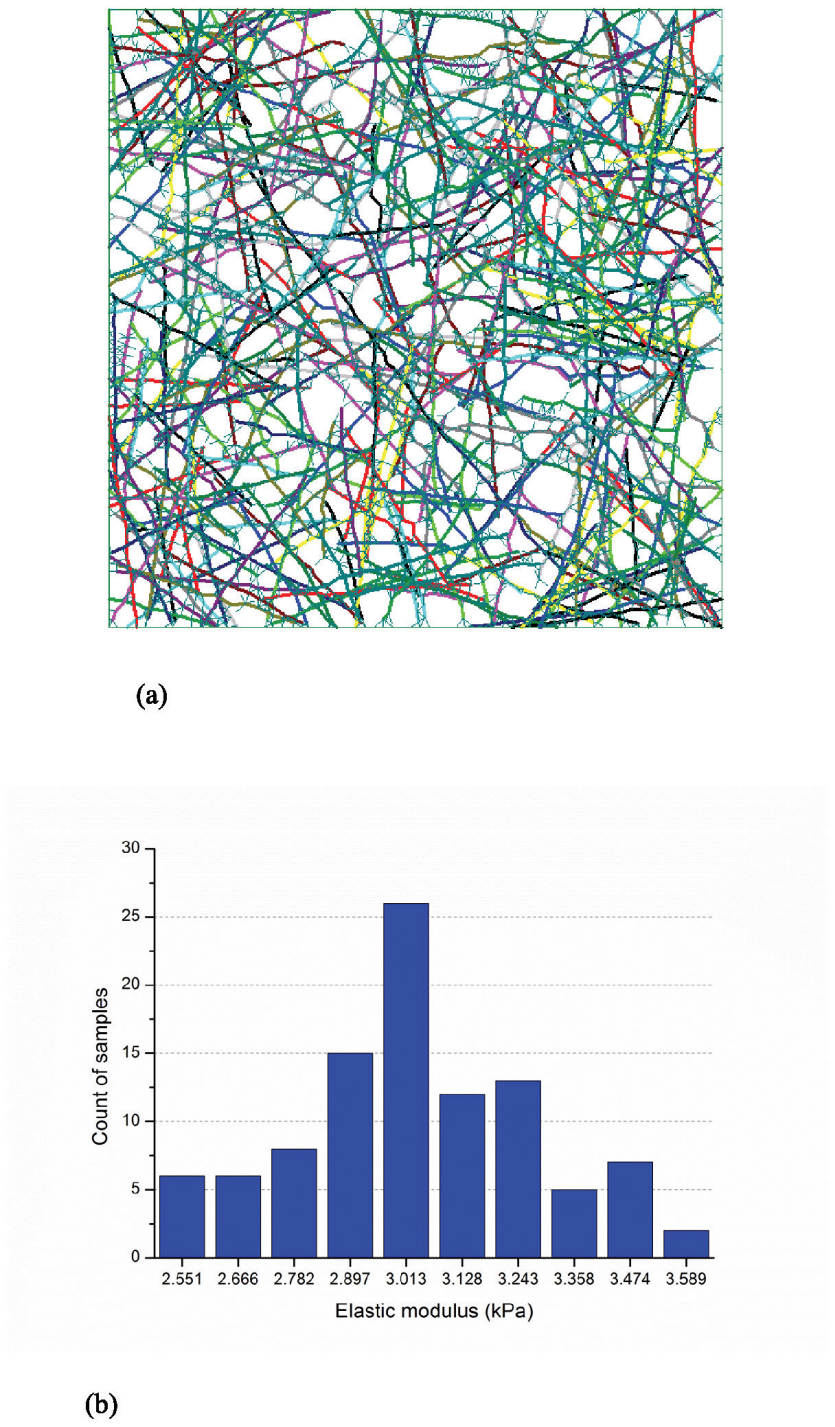
Typical model and response on 20μm×20μm domain (100 samples, actin filament relative density: 0.15%, actin filament length: 5μm±2μm, segment length 0.3μm±0.06μm, and maximum cross-linker length: 0.3 μm) (a) layout of a selected sample (20μm×20μm) (b) histogram of elastic moduli, $\bar{E}$=3.09kPa, std(E)=0.24kPa.

Elastic modulus of networks, with a relative density of 0.15%, was studied for domains of 10 μm × 10 μm and 20 μm × 20 μm. Typical network topology and histograms from the 100 samples of predicted elastic moduli for the two domain sizes are provided in Figure 5 and 6. Although the distributions of predicted elastic moduli in Figures 5(b) and 6(b) are modestly different, their mean values, 3.11 kPa and 3.09 kPa, are close and the 10 μm × 10 μm is deemed adequate. Note, here the fidelity of the form-finding model can be judged – the predictions are in the same order of elastic moduli as measured by reliable experimental techniques [1].

## Results

### Filaments orient under extension

Actin filaments re-orient when a cell is under mechanical stimulation. The exact nature of this re-orientation is complex and depends on the time-scales of the stimulation, the stiffness of the substrate employed in the stretching and a host of biological factors. At short time scales as the filament network responds to elongation filaments may align parallel to the stretch [28]; for other cases alignment may be perpendicular to the direction of stretch [29] unless specific proteins are knocked down[30]. The current form-finding analysis has been used to explore this phenomenon. 

**Figure 7:**
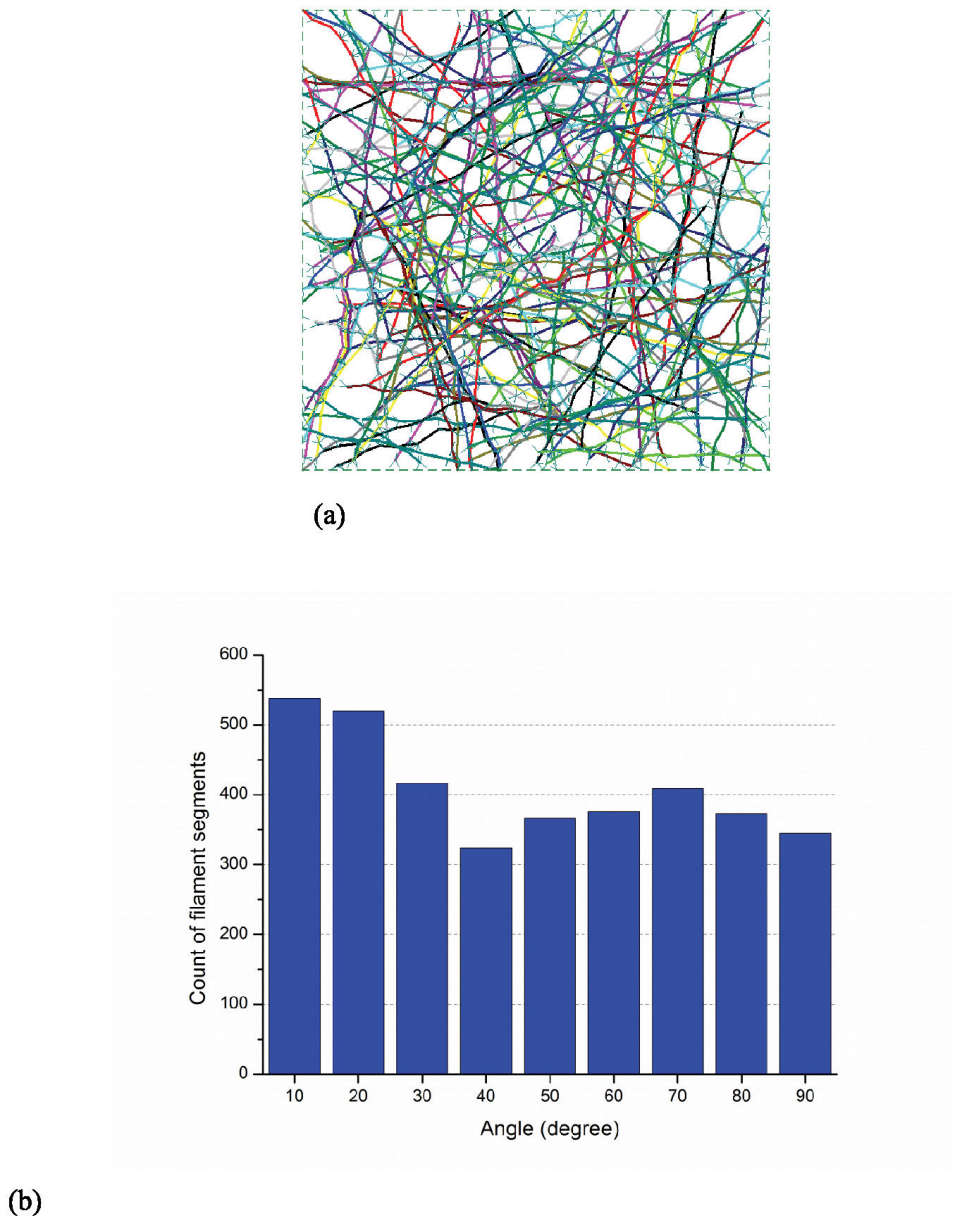
Initial layout and orientation of filament segments (a) initial layout of a selected sample (b) histogram of angles between filament segments and horizontal axis at the initial state.

**Figure 8:**
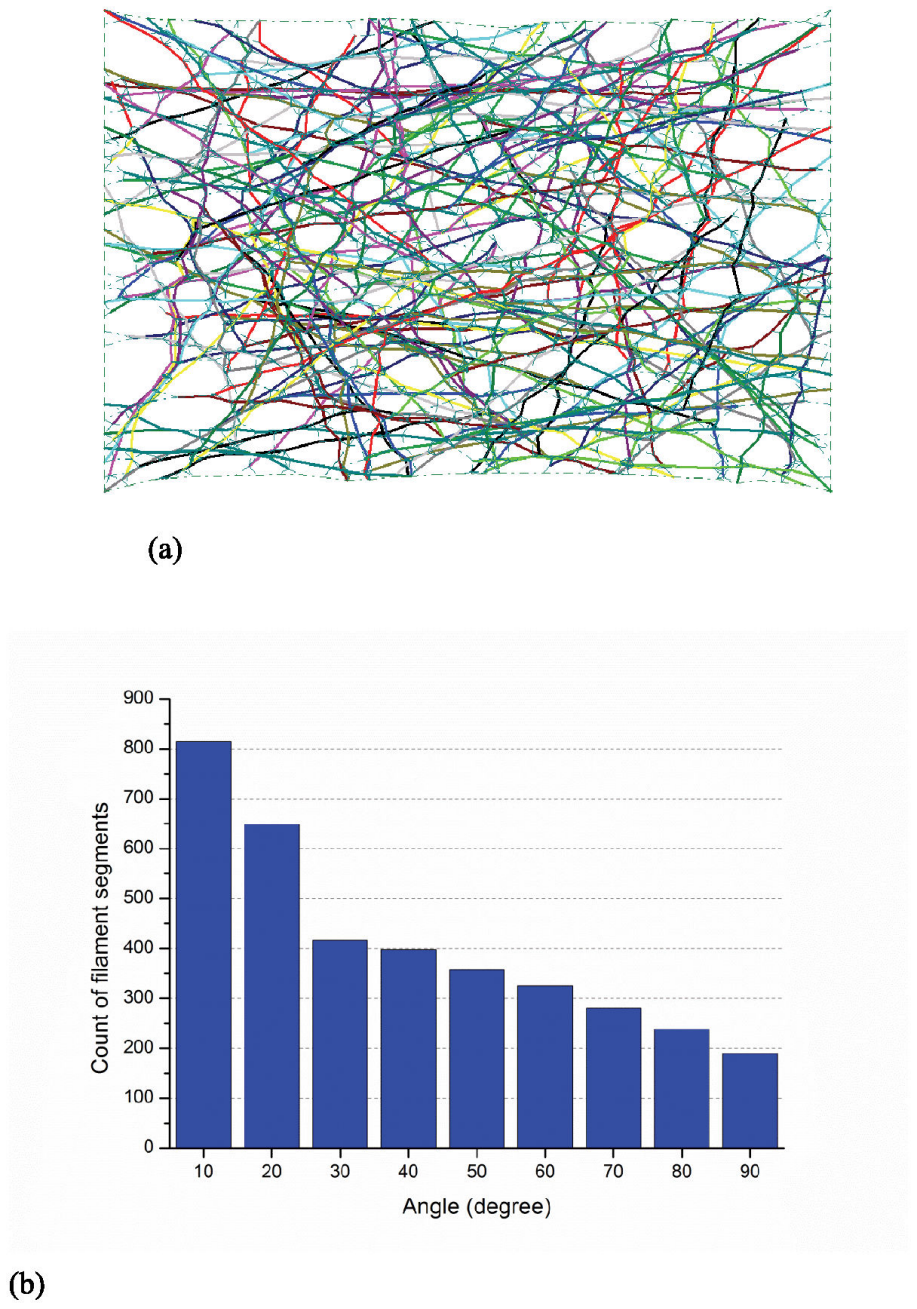
Deformed shape and orientation at 50% tensile strain (a) layout of a selected sample at 50% extension (b) histogram of angles between filament segments and horizontal axis.

**Figure 9:**
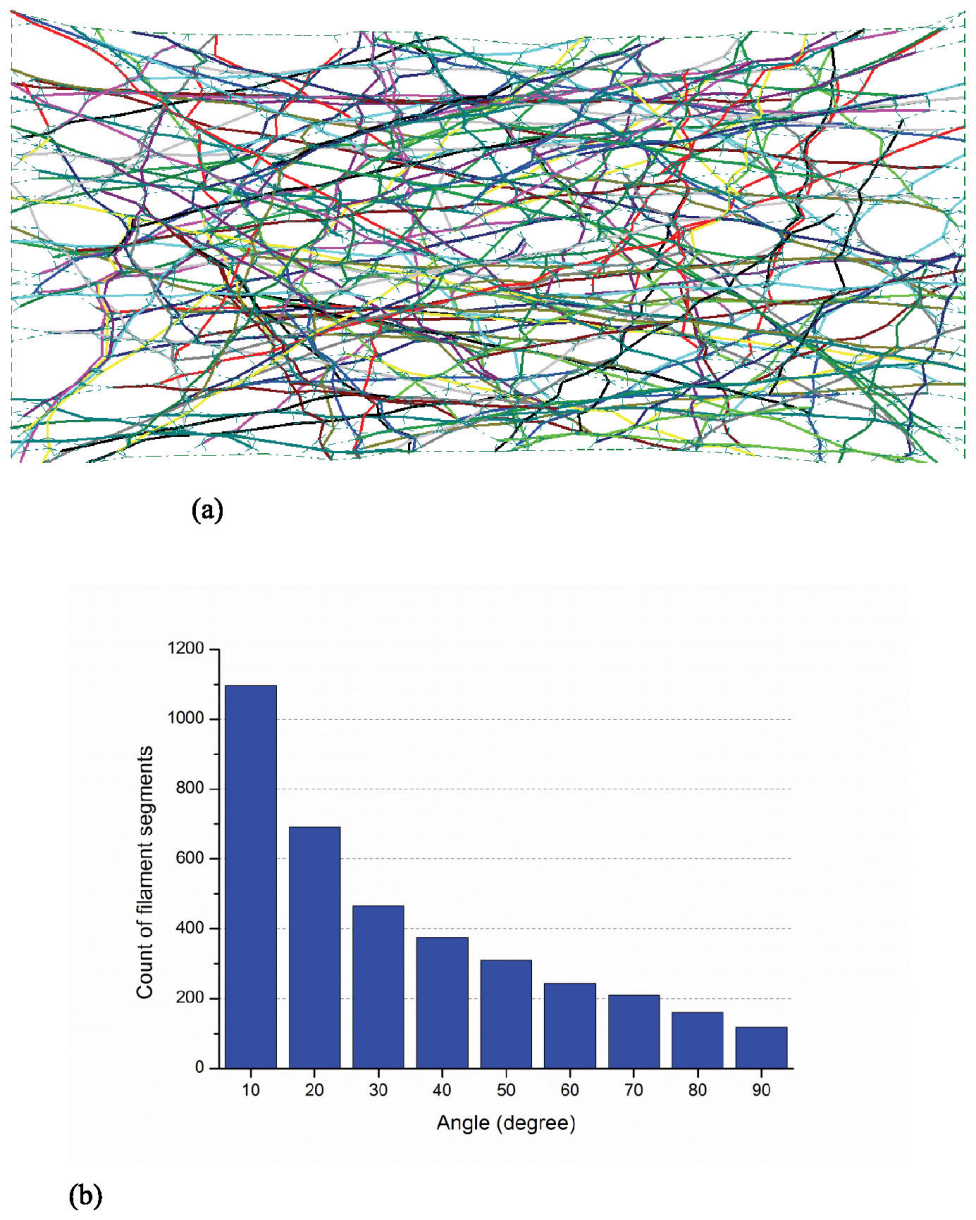
Deformed shape and orientation at 100% tensile strain (a) layout of a selected sample at 100% extension (b) histogram of angles between filament segments and horizontal axis.

The orientation of filament segments is quantified when the actin-network is under uniform extension (in the horizontal direction). Form-finding simulations consisting of 100 samples have been completed and the actin filament relative density is 0.25%. Figures 7-9 provide a typical sample and the histogram of filament segment orientation at strain levels of 0%, 50%, and 100%, respectively. In the initial state (Figure 7) the angular orientation for the filament segments is uniformly randomly distributed from 0° to 90°. However, as the network is stretched the filaments begin to exhibit a distinct alignment in the horizontal direction as they experience higher levels of straining, as detailed in Figures 8 and 9. Thus, the physics of the model demands parallel orientation of the filaments, and the model will have to have additional feedback included to exhibit the type of behavior observed in [29].

### Filament and filamin control network stiffness

The relative densities of actin filaments and actin-cross-linking proteins dramatically alter the stiffness of actin networks [31-36]. Moreover, actin filament length affects the rheology of these networks and therefore further “tunes” their elasticity [22]. The developed form-finding model enables us to directly investigate the effects of relative density of the filaments or cross-linkers, and filament length on the network stiffness.

#### Relative density of filaments positively correlated with cytoskeletal stiffness

**Figure 10:**
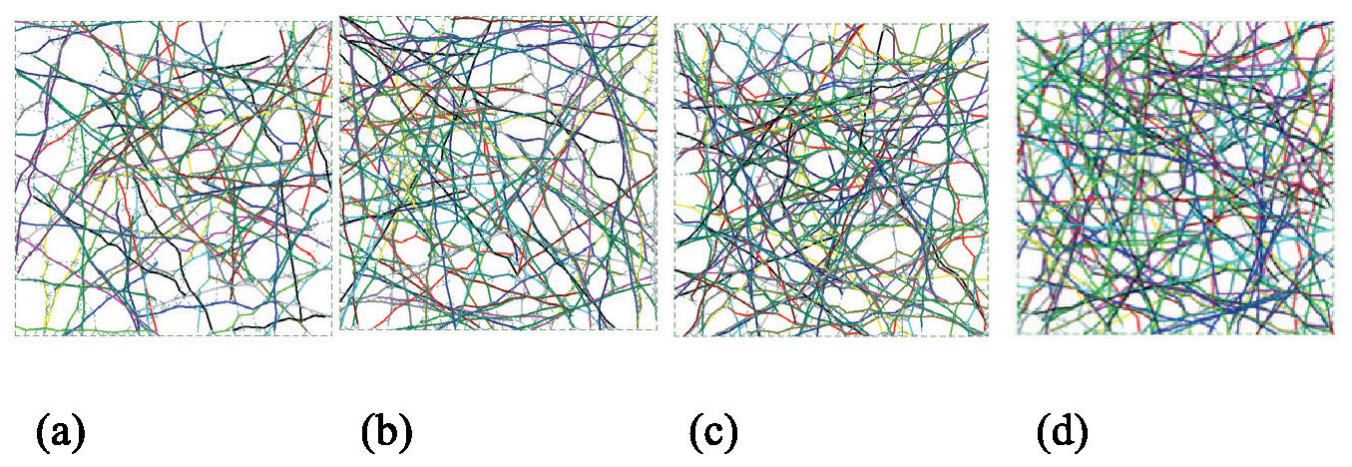
Layouts of selected samples with different actin filament relative densities. (a) 0.15% (b) 0.20% (c) 0.25% (d) 0.30%.

**Figure 11:**
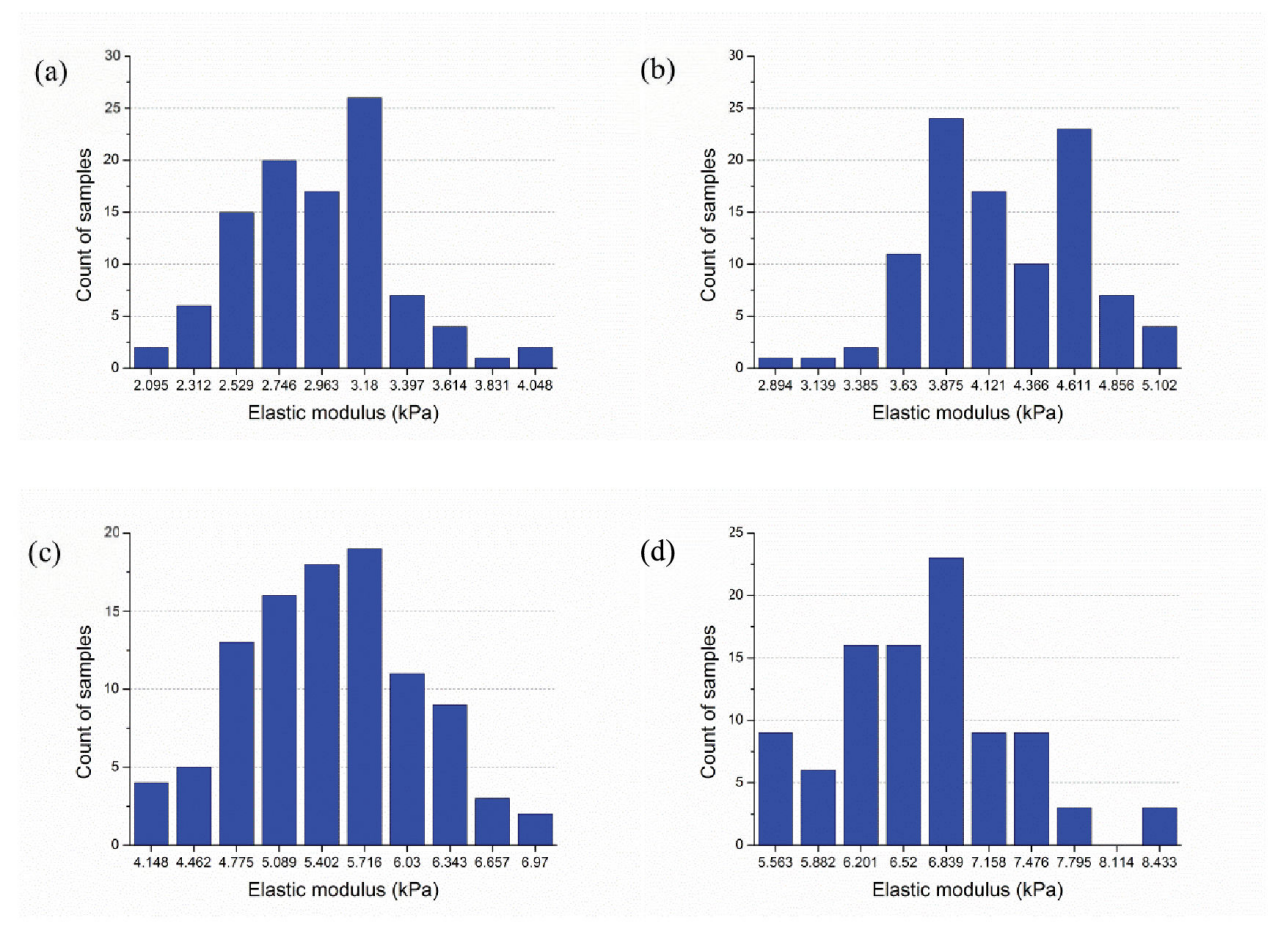
Impact of filament relative density on stiffness (a) filament relative density of 0.15%, $\bar{E}$=3.11kPa, std(E)=0.40kPa; (b) filament relative density of 0.20%, $\bar{E}$ = 4.38kPa, std(E) = 0.47kPa; (c) filament relative density of 0.25%, $\bar{E}$ = 5.69kPa, std(E) = 0.66kPa; (d) filament relative density of 0.30%, $\bar{E}$ = 6.83kPa, std(E) = 0.67kPa. $\bar{E}$and std(E) denote sample mean and standard deviation of elastic modulus E, respectively.

**Figure 12:**
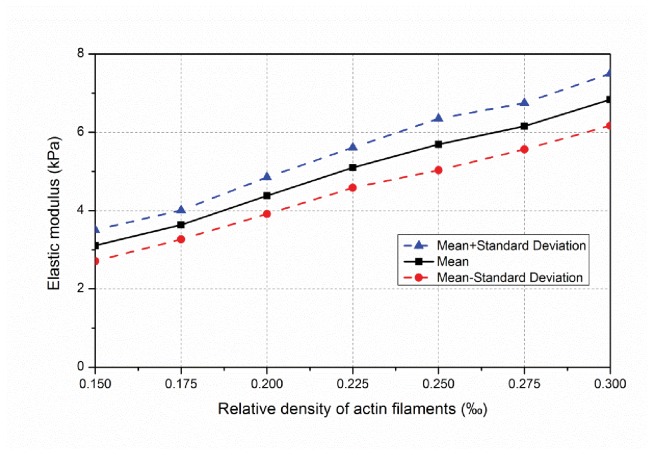
Variation of average stiffness of networks with relative density of actin filaments.

We used seven sets of samples (100 each), across filament relative densities of 0.15%, 0.175%, 0.20%, 0.225%, 0.25%, 0.275% and 0.30%. The topology of selected samples are provided in Figure 10 and show how relative density influences pore size and inter-connectedness of the developed network. At each relative density 100 samples are completed and the effective elastic modulus predicted. Histograms of the mean and standard deviation of elastic modulus for each filament are provided in Figure 11. Relative density plays a powerful role in determining network stiffness, e.g. the modulus is 3.11 kPa and 6.83 kPa at 0.15% and 0.30% relative density, respectively. The variation of mean network stiffness (modulus) with filament density is provided in Figure 12, where an approximately linear relation can be observed.

#### Filament length positively correlated with cytoskeletal stiffness

**Figure 13:**
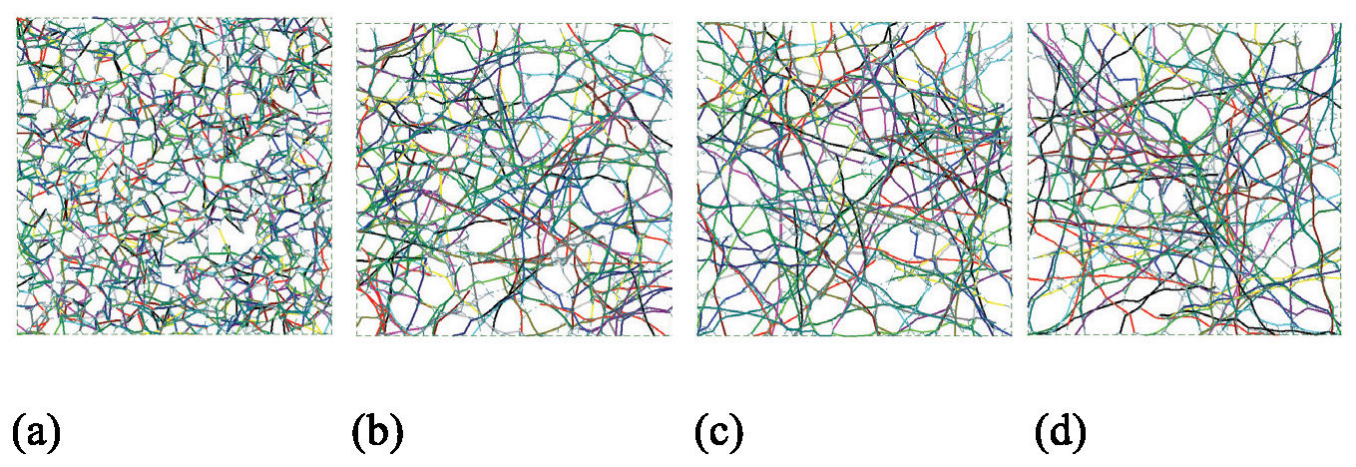
Layouts of selected samples having different lengths of actin filaments (a) 0.6±0.12μm (b) 2.4±0.48μm (c) 4.2±0.84μm (d) 6±1.2μm.

**Figure 14:**
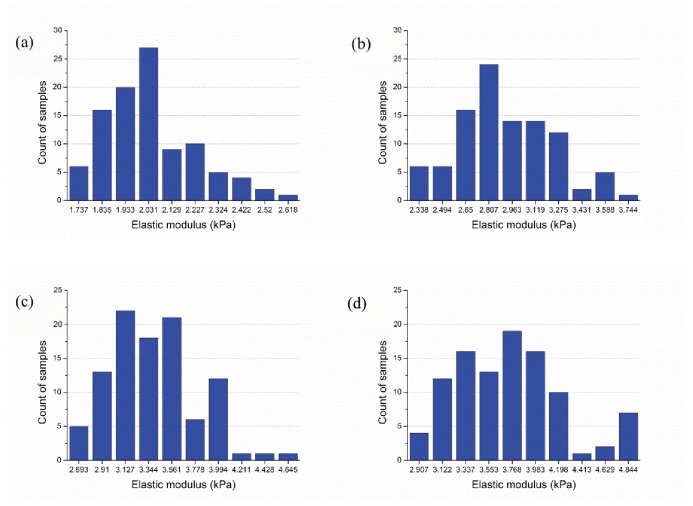
Impact of average actin filament length (L) on stiffness (filament relative density of 0.2%) (a) actin filament length L = 0.6±0.12μm, $\bar{E}$ = 2.08kPa, std(E) = 0.19kPa; (b) L = 2.4±0.48μm, $\bar{E}$ = 3.00kPa, std(E) = 0.32kPa; (c) L = 4.2±0.84μm, $\bar{E}$ = 3.50kPa, std(E) = 0.39kPa; (d) L = 6.0±1.2μm, $\bar{E}$ = 3.85kPa, std(E) = 0.49kPa.

Changing average filament length at a fixed relative density provides another means to alter the network topology (Figure 13) and resulting stiffness. A set of ten studies (100 samples in each study) is completed where the average and standard deviation of the filament length is varied - average filament length is varied from 0.6 μm to 6 μm in increments of 0.6 μm and the standard deviation is perfectly correlated to the length increasing from 0.12 μm up to 1.2 μm in increments of 0.12 μm. At the shortest filament length (Figure 13(a)) individual filaments typically only have one or two cross-linked connections to other filaments. At the longest filament length (Figure 13(d)) individual filaments have many, often more than 10, cross-linked connections to other filaments. This inter-connectivity (even at the same overall relative density) is rewarded with higher average effective modulus ($\bar{E}$): 3.85 kPa for the longest average filament length (Figure 13(d) and 14(d)) versus only 2.08 kPa for the shortest average filament length (Figure 13(a) and 14(a)). 

**Figure 15:**
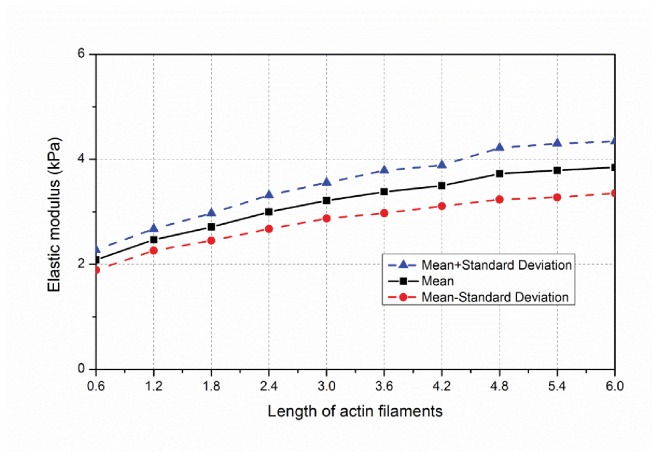
Variation of network stiffness as a function of average filament length.

Sample histograms for the resulting effective modulus are provided in Figure 14 and summary statistics across the full study in Figure 15. Note, at the longest filament length (6.0±1.2μm,Figure 13(d) and 14(d)) a larger number of high stiffness outliers result –suggesting that we are near the limit for our domain size and boundary effects are beginning to influence the statistics. Thus, longer filament lengths are not recommended for study without increasing the domain size. Taken in total, Figure 15 shows that actin filament length varies effective modulus by as much as nearly 2 kPa, similar to the total variation for studied relative density (Figure 12).

#### Relative density of cross-linkers positively correlated with cytoskeletal stiffness

**Figure 16:**
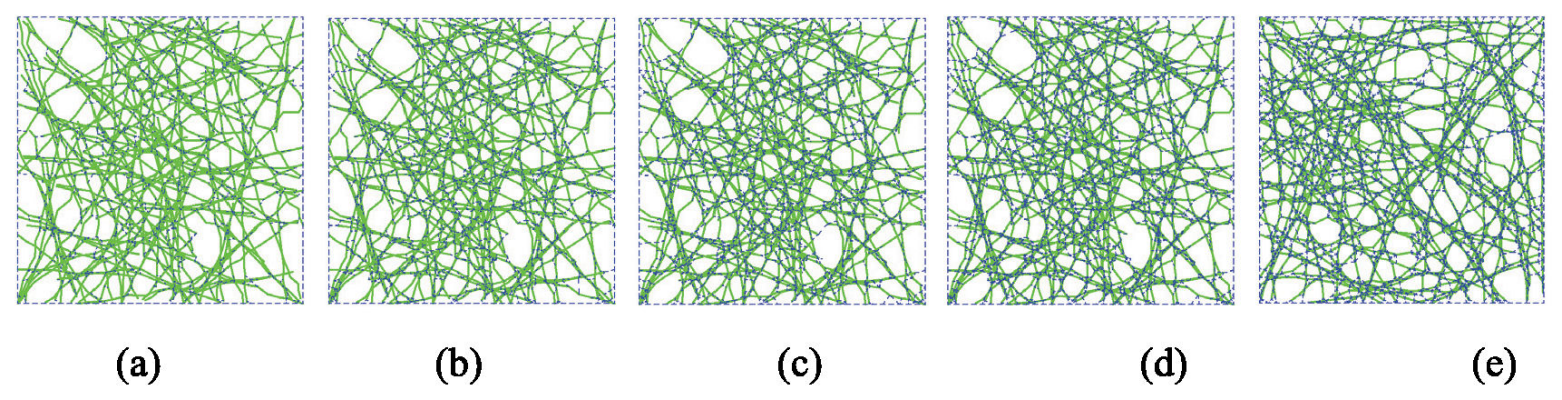
Layouts of selected samples having (a) 20%, (b) 40%, (c) 60%, (d) 80%, and (e) 100% cross-linkers. To highlight the cross-linkers, they are marked by blue dashed lines and actin filaments are in green.

**Figure 17:**
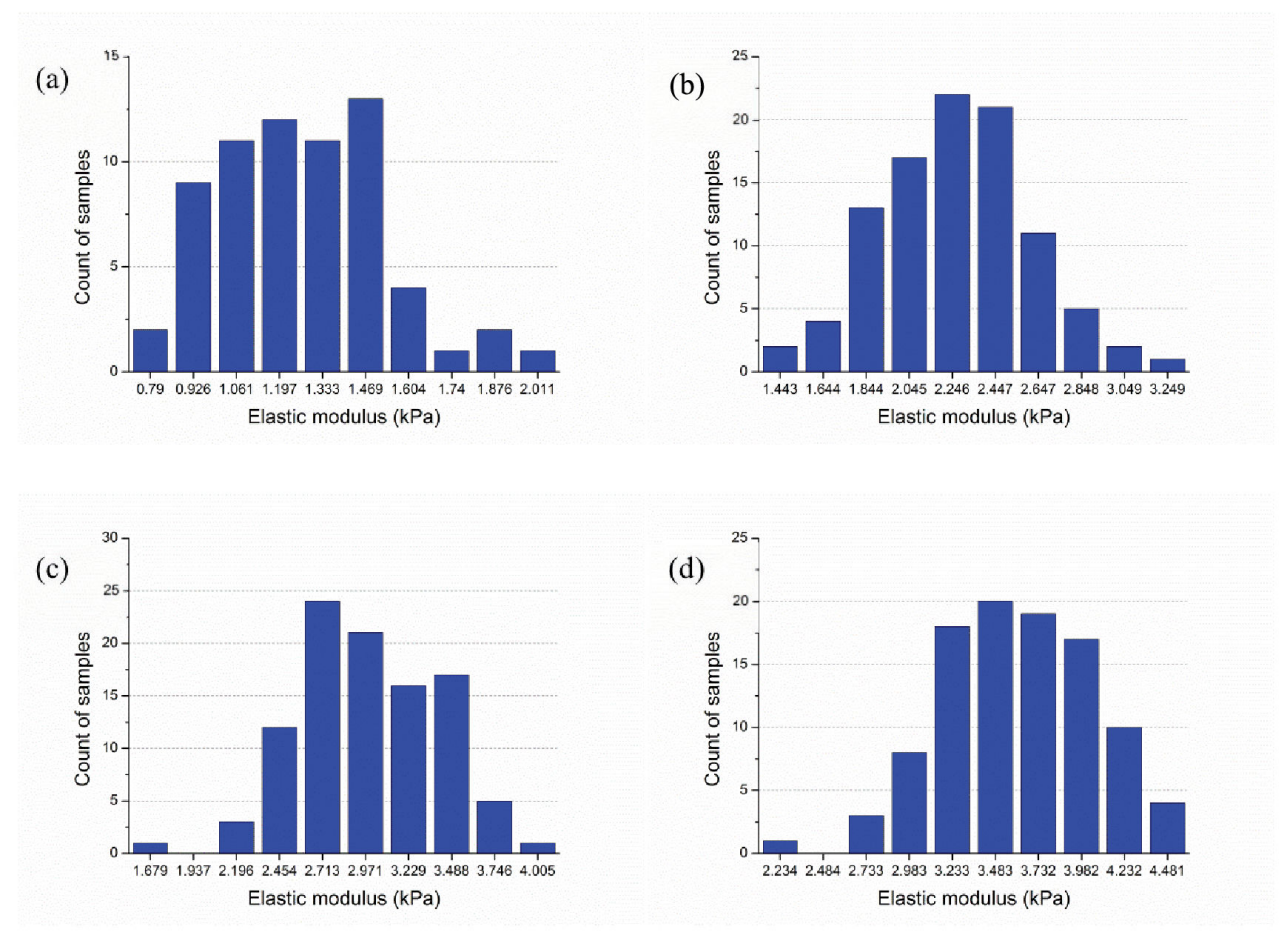
Impact of cross-linker density (f) on stiffness (filament relative density of 0.2): (a) cross-linker density f = 20%, $\bar{E}$ = 1.35 kPa, std(E) = 0.27kPa; (b) f = 40%, $\bar{E}$ = 2.39kPa, std(E) = 0.35kPa; (c) f = 60%, $\bar{E}$ = 3.15 kPa, std(E) = 0.42kPa; (d) f = 80%, $\bar{E}$ = 3.77kPa, std(E) = 0.46kPa.

**Figure 18:**
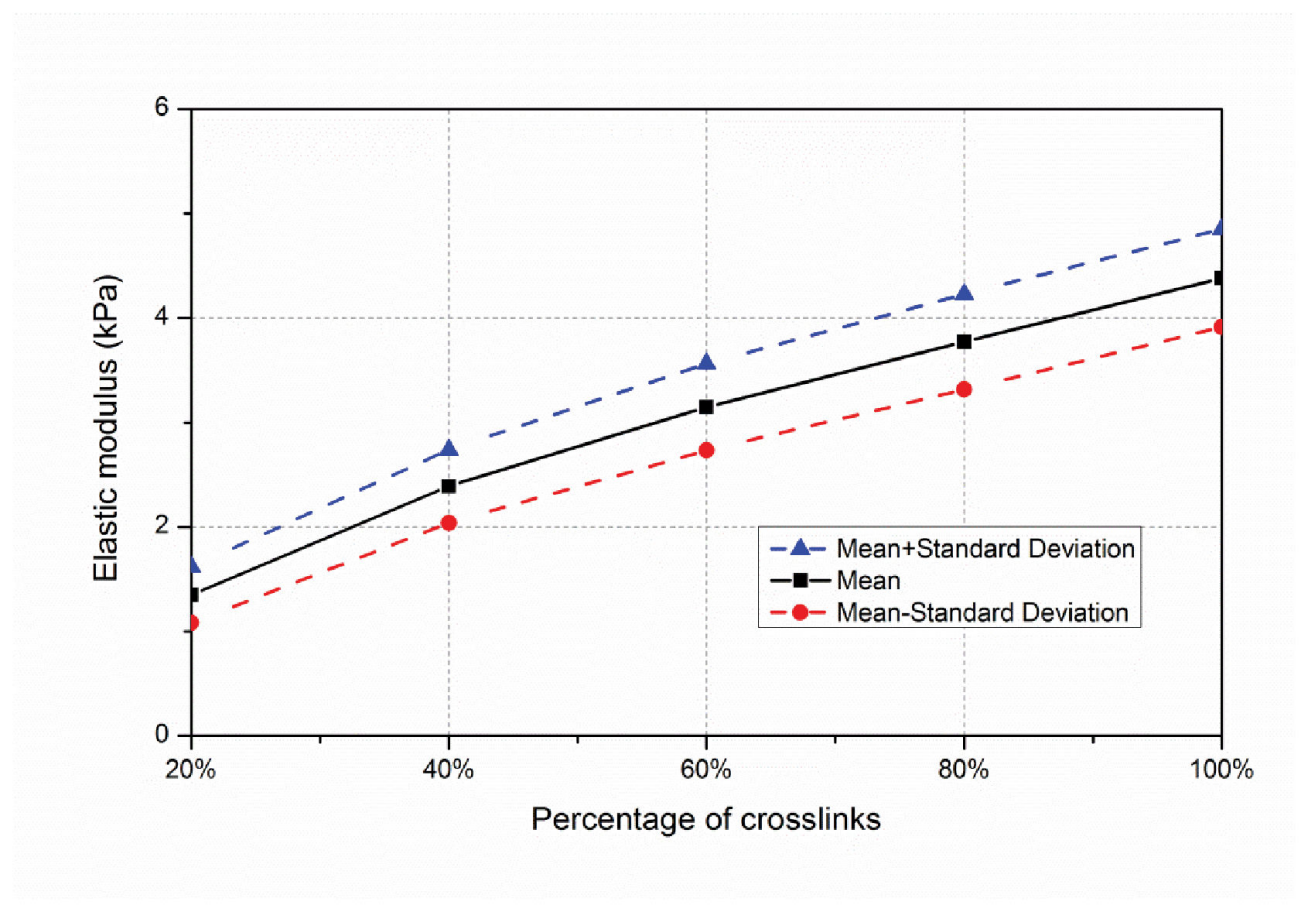
Variation of network stiffness with percentage of cross-linkers.

In the developed form-finding model the cross-linking protein connects the actin-network together. In the baseline model the actin filaments have host sites, which are 0.3 ± 0.06 μm apart (these are the segment ends, described in Section 2.1.4) and any two host sites that are less than 0.3 μm are connected by a cross-linker. Here, instead of connecting all host sites that are less than 0.3 μm only a percentage are randomly connected, varying from 20% up to 100% (baseline). For a given actin filament topology (a single network realization) the resulting cross-linking is depicted in Figure 16. Histograms of the effective modulus across 100 samples are provided for 20% to 80% cross-linking density in Figure 17 and the average ± a standard deviation are depicted for all results in Figure 18. Effective modulus increases by 3 kPa from 20% up to 100% cross-linking.

### Form-finding model provides basis for a more balanced approach

The open-cell foam model and the tensegrity model of cytoskeletal network stiffness are important idealizations, but they suffer fundamentally in that they assume that all the deformations are either bending (open-cell foam) or axial (tensegrity). In fact, no reason exists that this should be the case, and the form-finding model demonstrates how both modes of deformation play a role in the solution. 

**Figure 19:**
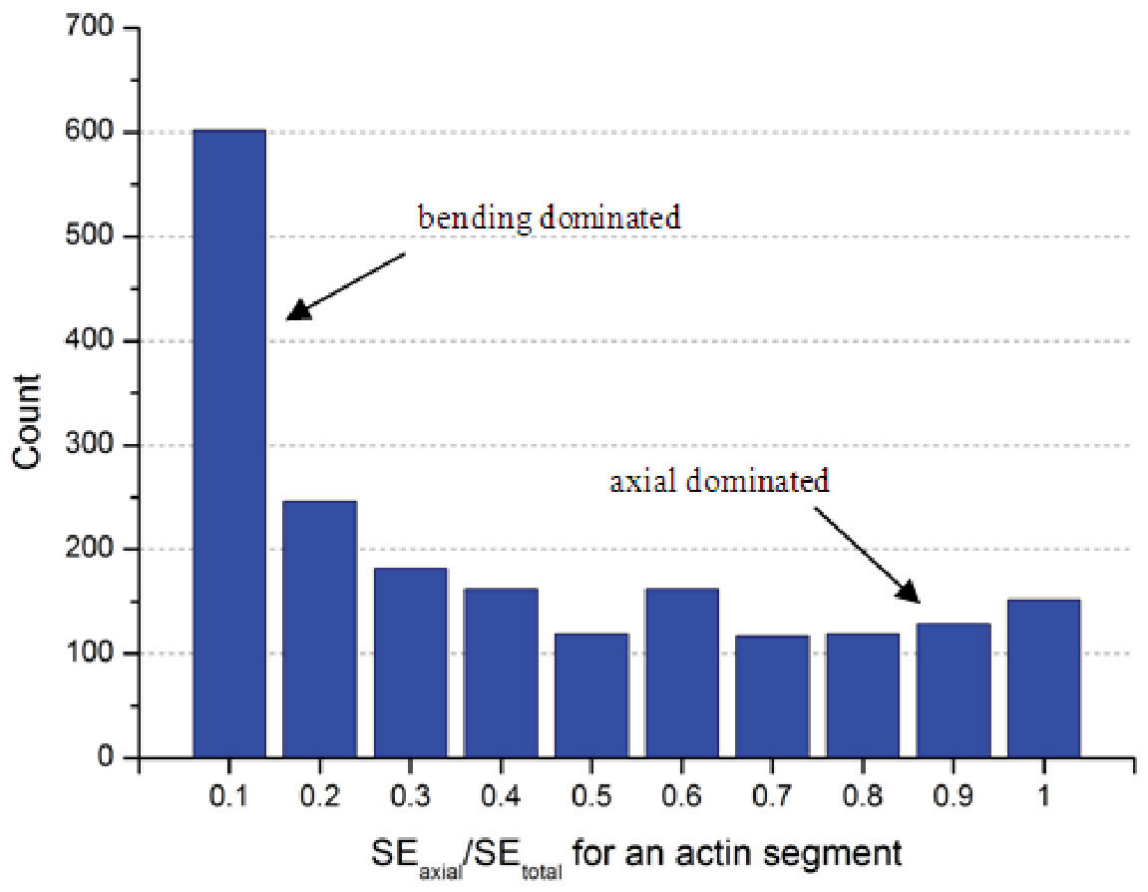
Histogram of actin filament energy fraction that is axial strain energy (10μm×10μm, actin filament relative density 0.15%, actin filament length 5μm±2μm, segment length 0.3±0.06μm, and maximum cross-linker length: 0.3μm).

For each actin filament, under deformation, we may determine the strain energy due to bending (SE_bending_) and the strain energy due to axial deformation (SE_axial_). The total strain energy (SE_total_) is the summation of these two. For our baseline model (0.15% relative density, further details in Table 1) a histogram of the fraction of axial strain energy (SE_axial_/SE_total_) is provided in Figure 19. As indicated in Figure 19, most of the actin filaments have very low axial strain energy and hence high bending strain energy. Hence the actin-network may be considered to be dominated by bending deformations and behave in a beam-like fashion. Nonetheless, a large number of actin filaments are dominated by axial (truss-like) deformations – and many more still are in between. The specifics of these results are sensitive to relative density, cross-linker density, mode of deformation, etc. Here, the form-finding model shows that both modes of deformation are necessary for realistic modeling of cytoskeletal network stiffness.

## Discussion

The developed form-finding model provides a means to generate realistic cytoskeletal network topologies with major biological features incorporated into the model. Instead of beginning from a set, ordered, topology as in existing models the form-finding model begins with a random array of materials. Order in the network is found by adhering to a fundamental law, in this case a stable equilibrium position, which is achieved through exploring large deformations in the network: i.e., through form-finding. Form-finding analysis, which is well known in designing tensile membrane structures (tents, etc.) within structural engineering, is well suited for the problem at hand.

A useful feature of the form-finding model is that it is inherently able to handle large deformations. The massive re-orientation of actin filament fibers that happens under stretching in one direction is readily included in the form-finding model as shown in Figure 9. Since most biologically relevant deformations are large in nature this ability is an essential feature for extensions to studies on cell morphology and motility.

The developed form-finding model demonstrates that relative density, actin filament length, and the concentration of actin-cross-linking protein, all play an influential role in the effective modulus of a cell. For the studied details relative density (varied from 0.15% to 0.30%) plays the strongest role, creating a variation of almost 4 kPa in stiffness across the studied range; however, variation in actin filament length created a 2 kPa spread, and variation in the concentration (presented by density) of cross-linker (filamin) created a 3 kPa spread. Thus, we come to the biologically relevant observation that, through determining the number of actin filaments (relative density), the length of actin filaments, and the degree to which they are cross-linked, the cell has fine-level control over the developed stiffness. Further, we note that these features are all captured in the form-finding model.

Given the many length and time scales inherent in cellular response multi-scale models are a natural long-term direction for model development. The form-finding model developed herein provides relevant features to cross scales. For example, at finer scales, actin-cross-linking protein binding sites on the actin filament are included in the model and detailed models of the cross-linking process could be included. At coarser scales, the overall effective modulus of a given realization of cytoskeletal network can readily be included in a continuum scale model. 

Significant future work remains to advance the form-finding model. The most important of which is the move from two-dimensions to three-dimensions. As the SEM of Figure 1(b) shows the network has depth and the filaments are clearly oriented in space, not just a plane. Initial work in this direction has been completed and it may be stated that stable equilibrium configurations in three-dimensions, using the same techniques for building up the form-finding model, exist. Model complexity and computational size quickly expand presenting certain challenges to large stochastic simulations, but it is felt by the authors that this direction has the most promise and is an active area of current research. Additional work utilizing the form-finding model to directly address cytoskeletal viscoelasticity is also needed. The form-finding model provides the framework for this study, but detailed time-dependent parameters of the filaments and cross-linkers is required.

## Conclusions

A stochastic form-finding model has been developed to study the stiffness of the cytoskeleton network. The developed two-dimensional model includes actin filaments and cross-linking proteins and is realized on a 10 μm × 10 μm domain. For a given random initial actin filament topology and density of cross-linkers a form-finding analysis is performed to generate a stable configuration for the network. The resulting network is topologically similar to real actin-networks. Through simulation of simple stretching the performance of the network is assessed. Both bending and axial deformations occur within the actin filaments. Consistent with actual cell response it is also shown how actin filaments align and re-orient under large stretching. The predicted effective modulus under stretching is consistent with experimental observations. The form-finding model also shows how effective modulus is sensitive to relative density, actin filament length, and cross-linker density. Work to extend the model to three dimensions is underway. Taken together, the form-finding model provides a new means for studying mechanical properties of cytoskeletal networks. 

## References

[1] Ethier CR, Simmons CA (2007) Introductory biomechanics: from cells to organisms. Cambridge Univ Pr.

[2] Pollard TD, Cooper JA (2009) Actin, a central player in cell shape and movement. Science 326: 1208–1212. doi:10.1126/science.1175862. PubMed: 19965462. 19965462 PMC3677050

[3] Satcher RL Jr, Dewey CF Jr (1996) Theoretical estimates of mechanical properties of the endothelial cell cytoskeleton. Biophys J 71: 109-118. doi:10.1016/S0006-3495(96)79206-8. PubMed: 8804594. 8804594 PMC1233462

[4] Ingber DE (1993) Cellular tensegrity: defining new rules of biological design that govern the cytoskeleton. J Cell Sci 104: 613-613. PubMed: 8314865. 8314865 10.1242/jcs.104.3.613

[5] Cañadas P, Laurent VM, Oddou C, Isabey D, Wendling S (2002) A cellular tensegrity model to analyse the structural viscoelasticity of the cytoskeleton. J Theor Biol 218: 155-173. doi:10.1006/jtbi.2002.3064. PubMed: 12381289. 12381289

[6] Baudriller H, Maurin B, Cañadas P, Montcourrier P, Parmeggiani A et al. (2006) Form-finding of complex tensegrity structures: application to cell cytoskeleton modelling. Comp R Mec 334: 662-668. doi:10.1016/j.crme.2006.08.004.

[7] Luo Y, Xu X, Lele T, Kumar S, Ingber DE (2008) A multi-modular tensegrity model of an actin stress fiber. J Biomech 41: 2379-2387. doi:10.1016/j.jbiomech.2008.05.026. PubMed: 18632107. 18632107 PMC2603623

[8] Coughlin MF, Stamenović D (2003) A prestressed cable network model of the adherent cell cytoskeleton. Biophys J 84: 1328-1336. doi:10.1016/S0006-3495(03)74948-0. PubMed: 12547813. 12547813 PMC1302709

[9] Broedersz CP, Mao X, Lubensky TC, MacKintosh FC (2011) Criticality and isostaticity in fibre networks. Nat Phys. 7: 983-988. doi:10.1038/nphys2127.

[10] Chu JW, Voth GA (2006) Coarse-grained modeling of the actin filament derived from atomistic-scale simulations. Biophys J 90: 1572-1582. doi:10.1529/biophysj.105.073924. PubMed: 16361345. 16361345 PMC1367308

[11] Kang J, Steward RL, Kim YT, Schwartz RS, LeDuc PR et al. (2011) Response of an actin filament network model under cyclic stretching through a coarse grained Monte Carlo approach. J Theor Biol.274(1): 109-119. doi:10.1016/j.jtbi.2011.01.011. PubMed: 21241710. 21241710 PMC3501734

[12] Kim T, Hwang W, Kamm R (2009) Computational analysis of a cross-linked actin-like network. Exp Mech 49: 91-104. doi:10.1007/s11340-007-9091-3.

[13] Kim T, Hwang W, Lee H, Kamm RD (2009) Computational analysis of viscoelastic properties of crosslinked actin networks. PLOS Comput Biol 5: e1000439 PubMed: 19609348. 19609348 10.1371/journal.pcbi.1000439 PMC2703781

[14] Ingber DE, Madri JA, Jamieson JD (1981) Role of basal lamina in neoplastic disorganization of tissue architecture. Proc Natl Acad Sci USA 78: 3901–3905. doi:10.1073/pnas.78.6.3901. PubMed: 7022458. 7022458 PMC319681

[15] Stamenović D, Coughlin MF (2000) A quantitative model of cellular elasticity based on tensegrity. J Biomech Eng 122: 39–43. doi:10.1115/1.429631. PubMed: 10790828. 10790828

[16] Wang N, Tolić-Nørrelykke IM, Chen J, Mijailovich SM, Butler JP et al. (2002) Cell prestress. I. Stiffness and prestress are closely associated in adherent contractile cells. Am J Physiol-Cell Physiol 282: C606-C616. doi:10.1152/ajpcell.00269.2001. PubMed: 11832346. 11832346

[17] Fernández P, Pullarkat PA, Ott A (2006) A master relation defines the nonlinear viscoelasticity of single fibroblasts. Biophys J 90: 3796-3805. doi:10.1529/biophysj.105.072215. PubMed: 16461394. 16461394 PMC1440760

[18] Lam RH, Weng S, Lu W, Fu J (2012) Live-cell subcellular measurement of cell stiffness using a microengineered stretchable micropost array membrane. Integr Biol 4: 1289-1298. doi:10.1039/c2ib20134h. PubMed: 22935822. PMC4088946 22935822

[19] Gardel ML, Nakamura F, Hartwig JH, Crocker JC, Stossel TP et al. (2006) Prestressed F-actin networks cross-linked by hinged filamins replicate mechanical properties of cells. Proc Natl Acad Sci U S A 103: 1762-1767. doi:10.1073/pnas.0504777103. PubMed: 16446458. 16446458 PMC1413620

[20] Mizuno D, Tardin C, Schmidt CF, MacKintosh FC (2007) Nonequilibrium mechanics of active cytoskeletal networks. Science 315: 370-373. doi:10.1126/science.1134404. PubMed: 17234946. 17234946

[21] Bausch A, Kroy K (2006) A bottom-up approach to cell mechanics. Nat Phys 2: 231-238. doi:10.1038/nphys260.

[22] Kasza KE, Broedersz CP, Koenderink GH, Lin YC, Messner W et al. (2010) Actin filament length tunes elasticity of flexibly cross-linked actin networks. Biophys J 99: 1091-1100. doi:10.1016/j.bpj.2010.06.025. PubMed: 20712992. 20712992 PMC2920742

[23] Ferrer JM, Lee H, Chen J, Pelz B, Nakamura F et al. (2008) Measuring molecular rupture forces between single actin filaments and actin-binding proteins. Proc Natl Acad Sci USA 105: 9221–9226. doi:10.1073/pnas.0706124105. PubMed: 18591676. 18591676 PMC2453742

[24] Furuike S, Ito T, Yamazaki M (2001) Mechanical unfolding of single filamin A (ABP-280) molecules detected by atomic force microscopy. FEBS Lett 498: 72-75. doi:10.1016/S0014-5793(01)02497-8. PubMed: 11389901. 11389901

[25] Sjöblom B, Salmazo A, Djinović-Carugo K (2008) a-Actinin structure and regulation. Cell Mol Life Sci 65: 2688-2701. doi:10.1007/s00018-008-8080-8. PubMed: 18488141. 18488141 PMC11131806

[26] Yamazaki M, Furuike S, Ito T (2002) Mechanical response of single filamin A (ABP-280) molecules and its role in the actin cytoskeleton. J Muscle Res Cell Motil 23: 525-534. doi:10.1023/A:1023418725001. PubMed: 12785102. 12785102

[27] Wolosewick JJ, Porter KR (1979) Microtrabecular lattice of the cytoplasmic ground substance. Artifact or reality. J Cell Biol 82: 114-139. doi:10.1083/jcb.82.1.114. PubMed: 479294. 479294 PMC2110423

[28] Walcott S, Sun SX (2010) A mechanical model of actin stress fiber formation and substrate elasticity sensing in adherent cells. Proc Natl Acad Sci USA 107: 7757-7762. doi:10.1073/pnas.0912739107. PubMed: 20385838. 20385838 PMC2867880

[29] Kaunas R, Nguyen P, Usami S, Chien S (2005) Cooperative effects of Rho and mechanical stretch on stress fiber organization. Proc Natl Acad Sci U S A 102: 15895-15900. doi:10.1073/pnas.0506041102. PubMed: 16247009. 16247009 PMC1276069

[30] Niediek V, Born S, Hampe N, Kirchgeßner N, Merkel R et al. (2011) Cyclic stretch induces reorientation of cells in a Src family kinase-and p130Cas-dependent manner. Eur J Cell Biol.91(2): 118-128. PubMed: 22178114. 22178114 10.1016/j.ejcb.2011.10.003

[31] Janmey PA, Hvidt S, Lamb J, Stossel TP (1990) Resemblance of actin-binding protein/actin gels to covalently crosslinked networks. Nature 345: 89 - 92. doi:10.1038/345089a0. PubMed: 2158633. 2158633

[32] Ruddies R, Goldmann WH, Isenberg G, Sackmann E (1993) The viscoelasticity of entangled actin networks: the influence of defects and modulation by talin and vinculin. Eur Biophys J 22: 309-321. doi:10.1007/BF00213554. PubMed: 8112218. 8112218

[33] Wachsstock DH, Schwarz WH, Pollard TD (1994) Cross-linker dynamics determine the mechanical properties of actin gels. Biophys J 66: 801-809. doi:10.1016/S0006-3495(94)80856-2. PubMed: 8011912. 8011912 PMC1275778

[34] Tempel M, Isenberg G, Sackmann E (1996) Temperature-induced sol-gel transition and microgel formation in α-actinin cross-linked actin networks: a rheological study. Phys Rev E 54: 1802. doi:10.1103/PhysRevE.54.1802. 9965260

[35] Xu J, Wirtz D, Pollard TD (1998) Dynamic cross-linking by α-actinin determines the mechanical properties of actin filament networks. J Biol Chem 273: 9570–9576. doi:10.1074/jbc.273.16.9570. PubMed: 9545287. 9545287

[36] Gardel ML, Shin JH, MacKintosh FC, Mahadevan L, Matsudaira P et al. (2004) Elastic behavior of cross-linked and bundled actin networks. Science 304: 1301-1305. doi:10.1126/science.1095087. PubMed: 15166374. 15166374

